# Crystal structure and luminescence spectrum of a one-dimensional nickel(II) coordination polymer incorporating 1,4-bis­[(2-methyl­imidazol-1-yl)meth­yl]benzene and adamantane-1,3-di­carboxyl­ate co-ligands

**DOI:** 10.1107/S2056989023006059

**Published:** 2023-07-14

**Authors:** Yong Zhang, Hongni Qin, Bing Wu

**Affiliations:** a Suzhou Industrial Park Institute of Services Outsourcing, Suzhou 215123, Jiangsu, People’s Republic of China; b College of Chemistry, Chemical Engineering and Materials Science, Soochow University, Suzhou 215123, People’s Republic of China; University of Aberdeen, United Kingdom

**Keywords:** crystal structure, nickel, coordination polymer

## Abstract

An Ni^II^ coordination polymer, namely, poly[(μ_2_-adamantane-1,3-di­carboxyl­ato-κ^4^ *O* ^1^,*O* ^1′^:*O* ^3^,*O* ^3′^)[μ_2_-1,4-bis­(2-methyl-imidazol-1-ylmeth­yl)benzene-κ^2^ *N* ^3^:*N* ^3′^]nickel(II)], [Ni(C_12_H_14_O_4_)(C_16_H_18_N_4_)]_*n*_ or [Ni(adc)(bmib)]_*n*_, (I) [adc = adamantane-1,3-di­carboxyl­ate, C_12_H_14_O_4_^2–^ and bmib = 1,4-bis­(2-methyl-imidazol-1-ylmeth­yl)benzene, C_16_H_18_N_4_] was synthesized and characterized. It exhibits a one-dimensional extended structure built up from alternating [Ni_2_(bmib)_2_] 26-membered rings and [Ni_2_(adc)_2_] 16-membered rings. The nickel atom lies on a crystallographic twofold axis and both ligands are completed by mirror symmetry. The solid-state luminescence spectra of (I) and the bmib ligand show strong emissions at 442 and 410 nm, respectively.

## Abstract 2

The title one-dimensional coordination polymer features alternating 26- and 16-membered rings.

## 1. Chemical context

Coordination polymers have been widely studied because of their diverse and inter­esting structures (Bao *et al.*, 2019 ▸; Zhang & Lin 2014 ▸; Wang *et al.*, 2020 ▸; Parmar *et al.*, 2021 ▸) and potential applications in sorption (Fan *et al.*, 2021 ▸), luminescent materials (Zhou *et al.*, 2021 ▸), magnetism (Yang *et al.*, 2021*a* ▸), catalytic splitting of water (Li *et al.*, 2019 ▸), catalytic degrading of pollutants (Jiang *et al.*, 2018 ▸) and battery mat­erials (Yang *et al.*, 2021*b* ▸; Bao *et al.*, 2019 ▸). In the construction of coordination polymers, N-donor (imidazole or triazole ligands) and O-donor (polycarboxyl­ate ligands) co-ligand systems lead to various inter­esting networks (Yang *et al.*, 2014 ▸; Sun *et al.*, 2013 ▸; Zhang *et al.*, 2021*a* ▸,*b* ▸). 1,4-Bis(2-methyl-imidazol-1-ylmeth­yl)benzene (C_16_H_18_N_4_; bmib) is a semi-flexible bidentate N-donor ligand and is widely used in the construction of different coordination polymers (Yang *et al.*, 2014 ▸; Sun *et al.*, 2013 ▸). Four Ni-bmib coordination polymers are documented: [Ni(bcpb)(bmib)_0.5_]_*n*_ (H_2_bcpb = 3,5-bis­(4-carb­oxy­phen­yl)pyridine) has a (3,4)-connected three-dimensional amd network, with the point symbol of (6^2.^8)(6^3.^8^.^10^2^) (Fan *et al.*, 2014*a* ▸). {[Ni(tptc)_0.5_(bmib)]·0.25H_2_O}_*n*_ (H_4_tptc = terphenyl-2,5,2′,5′-tetra­carb­oxy­lic acid) shows a (4,4)-coord­inated three-dimensional network with a point symbol of (4^.^6^4.^8^2^)_2_(4^2.^8^4^) (Fan *et al.*, 2014*b* ▸). [Ni(bmib)(bpda)] (H_2_bpda = biphenyl-3,4′-di­carb­oxy­lic acid) exhibits a threefold inter­penetrated (6^5.^8) network (Sun *et al.*, 2013 ▸). {[Ni_2_(glu)_2_(bmib)_2_(H_2_O)_2_]·H_2_O}_n_ (glu = glutarate) exhibits a 4-connected three-dimensional framework with point symbol 6^6^, but is not a typical dia network (Zhao *et al.*, 2020 ▸). The adamantane-1,3-di­carboxyl­ate dianion (C_12_H_14_O_4_^2–^; adc) is a good O-donor bridging ligand for constructing coordination polymers (Zhao *et al.*, 2017 ▸). In this work, the title Ni^II^ coordination polymer [Ni(adc)(bmib)]_*n*_, (I), was synthesized and its crystal structure was determined. 
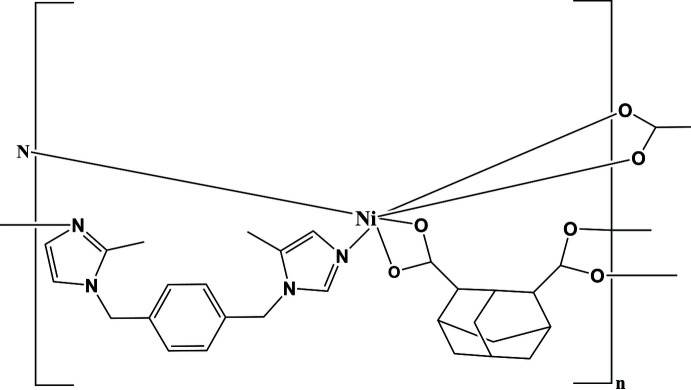


## 2. Structural commentary

**Figure 1:**
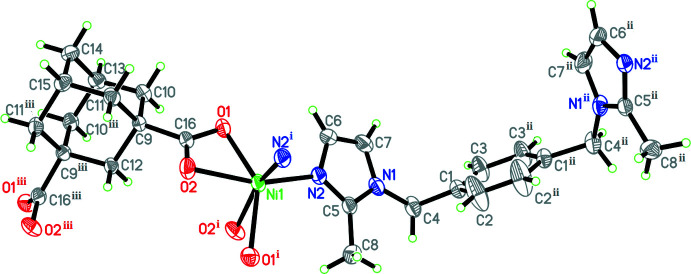
A view of the title compound with displacement ellipsoids drawn at the 50% probability level. Symmetry codes: (i) −*x* + 1, *y*, −*z*; (ii) *x*, −*y* + 1, *z*; (iii) *x*, −*y*, *z*.

**Figure 2:**
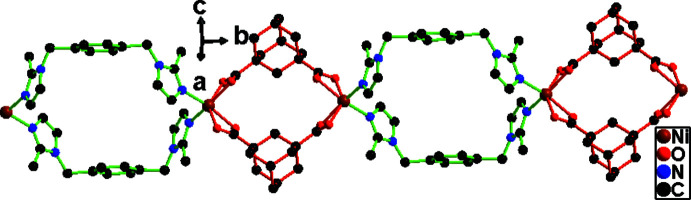
The one-dimensional supra­molecular structure of (I).

The structural motif of the title coordination polymer (I) is a one-dimensional chain. The Ni^II^ atom in (I) lies on a crystallographic twofold axis and adopts a distorted *cis*-NiN_2_O_4_ octa­hedral coordination geometry arising from four oxygen atoms from two carboxyl­ate groups in two adc ligands [Ni1—O1 = 2.179 (3) Å; Ni1—O2 = 2.096 (3) Å] and two nitro­gen atoms of two bmib ligands [Ni1—N2 = 2.050 (3) Å] (Table 1 ▸, Fig. 1 ▸). Atoms O1 and O1^i^ lie opposite to each other with the bond angle O1—Ni1—O1^i^ [symmetry code: (i) 1 – *x*, *y*, –*z*] = 142.26 (15)°. These Ni—O and Ni—N bond lengths are typical and show no deviations from those in other distorted octa­hedral Ni^II^ coordination polymers (Fan *et al.*, 2014*a* ▸,*b* ▸). The other bond angles are in the range 61.20 (11)–156.75 (13)° (Fan *et al.*, 2014*a* ▸,*b* ▸). The dihedral angle between the imidazole and benzene rings of the bmib mol­ecule is 78.8 (2)° and that between the imidazole rings is 67.1 (2)°. The bmib ligand exhibits a *gauche* conformation and the torsion angle N1—C4—C1—C3 is −117.9 (5)°. In the extended structure, two bmib ligands bridge two Ni^II^ atoms and construct a [Ni_2_(bmib)_2_] 26-membered ring with an Ni⋯Ni distance of 12.100 (2) Å. Two carboxyl­ate groups of one adc ligand exhibit an *O*,*O*-chelating mode such that two adc ligands link two Ni^II^ atoms and construct an [Ni_2_(adc)_2_] 16-membered ring with Ni⋯Ni = 8.0978 (16) Å. The Ni^II^ atoms are alternately connected by the bridging bmib and adc moieties, resulting in a chain containing alternative [Ni_2_(bmib)_2_] and [Ni_2_(adc)_2_] loops propagating along the *b*-axis direction (Fig. 2 ▸).

## 3. Supra­molecular features

**Figure 3:**
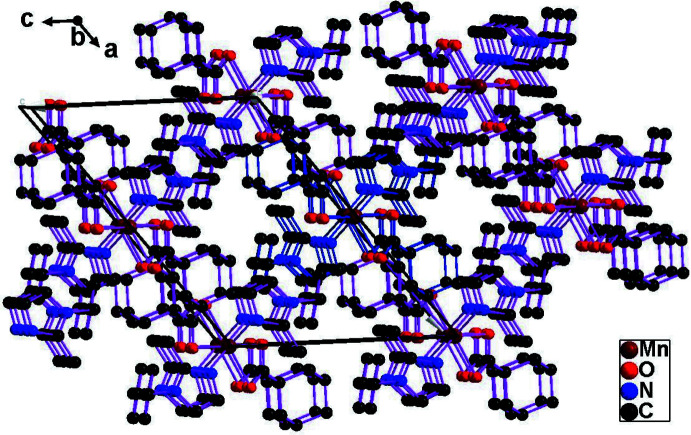
The stacking of [010] chains in the crystal structure of (I). The bonds of one chain are shown in blue and the bonds of six adjacent chains are shown in purple.

Each [Ni(bmib)(adc)]_n_ chain is surrounded by six further chains (Fig. 3 ▸). There are no C—H⋯O hydrogen bond inter­actions or aromatic π–π stacking inter­actions between the rings, thus the three-dimensional supra­molecular architecture of (I) must therefore be established by van der Waals inter­actions.

## 4. Luminescence properties

**Figure 4:**
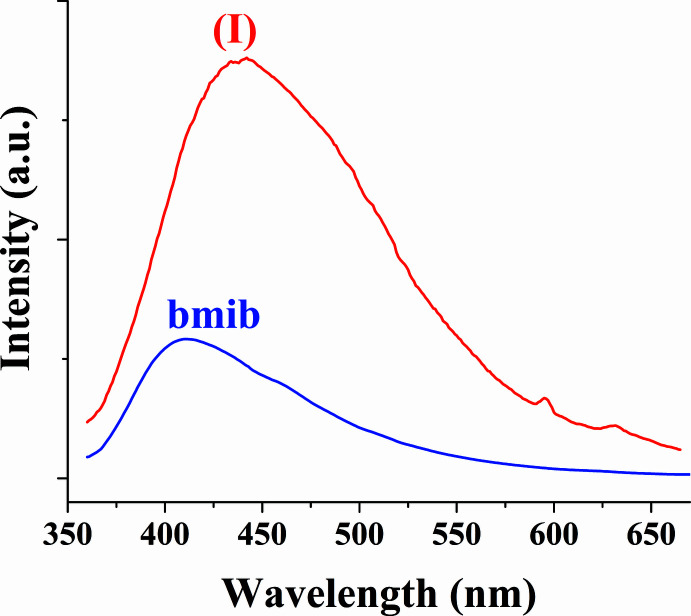
Solid-state luminescence spectra of (I) and the bmib ligand at room temperature.

The solid-state luminescence spectra of (I) and the bmib ligand were measured at room temperature (Fig. 4 ▸). Compound (I) and bmib exhibit strong emissions at 442 nm and 410 nm, respectively, upon excitation at 340 nm. The emissions can be attributed to an intra­ligand charge-transfer transition (Yang *et al.*, 2014 ▸).

## 5. Database survey

The bmib ligand is widely used in coordination chemistry but for Ni–bmib compounds, a search of the Cambridge Structural Database (CSD, version 5.42, update of September 2021; Groom *et al.*, 2016) revealed only the four coordination polymers noted in the *Chemical context* section.

## 6. Synthesis and crystallization

A mixture of bmib (0.22 mmol), Ni(NO_3_)_2_^.^6H_2_O (0.28 mmol), H_2_adc (0.22 mmol), NaOH (0.38 mmol) and H_2_O (14.0 ml) was added to a 20.0 ml Teflon-lined stainless steel autoclave, which was then sealed and heated to 393 K for 5 d. Green crystals of (I) were obtained when the mixture was cooled to room temperature.

## 7. Refinement

Crystal data, data collection and structure refinement details are summarized in Table 2 ▸. The hydrogen atoms (CH, CH_2_, CH_3_ groups) were placed geometrically (C—H = 0.93–0.98 Å) and refined using a riding model with *U*_iso_(H) = 1.2*U*_eq_(C) for CH and CH_2_ or 1.5*U*_eq_(C) for CH_3_ groups.

## Supplementary Material

Crystal structure: contains datablock(s) . DOI: 10.1107/S2056989023006059/hb8041sup1.cif

Structure factors: contains datablock(s) I. DOI: 10.1107/S2056989023006059/hb8041Isup3.hkl

CCDC reference: 2280450

Additional supporting information:  crystallographic information; 3D view; checkCIF report

## Figures and Tables

**Table 1:** Selected geometric parameters (Å, °)

Ni1—O1	2.179 (3)	Ni1—N2	2.050 (3)
Ni1—O2	2.096 (3)		
			
O1—Ni1—O1^i^	142.26 (15)	N2—Ni1—O1	95.55 (12)
O2—Ni1—O1	61.20 (11)	N2—Ni1—O2^i^	91.42 (13)
O2—Ni1—O1^i^	91.22 (12)	N2—Ni1—O2	156.75 (13)
O2—Ni1—O2^i^	89.19 (18)	N2—Ni1—N2^i^	97.01 (19)
N2—Ni1—O1^i^	109.38 (12)		

Symmetry code: (i) 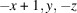 .

**Table 2:** Experimental details

Crystal data	
Chemical formula	[Ni(C_12_H_14_O_4_)(C_16_H_18_N_4_)]
*M*_r_	547.28
Crystal system, space group	Monoclinic, *C*2/*m*
Temperature (K)	293
*a*, *b*, *c* (Å)	14.489 (3), 20.198 (4), 10.741 (2)
β (°)	127.46 (3)
*V* (Å^3^)	2495.2 (12)
*Z*	4
Radiation type	Mo *K*α
μ (mm^−1^)	0.82
Crystal size (mm)	0.60 × 0.20 × 0.10

Data collection	
Diffractometer	Rigaku Mercury CCD
Absorption correction	Multi-scan (Jacobson, 1998 ▸)
*T*_min_, *T*_max_	0.639, 0.922
No. of measured, independent and observed [*I* > 2σ(*I*)] reflections	12149, 2343, 1975
*R*_int_	0.060
(sin θ/λ)_max_ (Å^−1^)	0.602

Refinement	
*R*[*F* ^2^ > 2σ(*F* ^2^)], *wR*(*F* ^2^), *S*	0.065, 0.148, 1.15
No. of reflections	2343
No. of parameters	174
H-atom treatment	H-atom parameters constrained
Δρ_max_, Δρ_min_ (e Å^−3^)	0.39, −0.42

Computer programs: *CrysAlis PRO* (Rigaku OD, 2021 ▸), *OLEX2.solve* (Bourhis *et al.*, 2015 ▸), *SHELXL* (Sheldrick, 2015 ▸) and *OLEX2* (Dolomanov *et al.*, 2009 ▸).

## supplementary crystallographic information

### Crystal data

**Table:**

[Ni(C_12_H_14_O_4_)(C_16_H_18_N_4_)]	*F*(000) = 1152
*M**_r_* = 547.28	*D*_x_ = 1.457 Mg m^−^^3^
Monoclinic, *C*2/*m*	Mo *K*α radiation, λ = 0.71073 Å
*a* = 14.489 (3) Å	Cell parameters from 4170 reflections
*b* = 20.198 (4) Å	θ = 3.1–25.3°
*c* = 10.741 (2) Å	µ = 0.82 mm^−^^1^
β = 127.46 (3)°	*T* = 293 K
*V* = 2495.2 (12) Å^3^	Block, green
*Z* = 4	0.60 × 0.20 × 0.10 mm

### Data collection

**Table:**

Rigaku Mercury CCD diffractometer	2343 independent reflections
Graphite monochromator	1975 reflections with *I* > 2σ(*I*)
Detector resolution: 7.31 pixels mm^-1^	*R*_int_ = 0.060
ω scans	θ_max_ = 25.4°, θ_min_ = 3.1°
Absorption correction: multi-scan (Jacobson, 1998)	*h* = −17→17
*T*_min_ = 0.639, *T*_max_ = 0.922	*k* = −20→24
12149 measured reflections	*l* = −12→12

### Refinement

**Table:**

Refinement on *F*^2^	Primary atom site location: iterative
Least-squares matrix: full	Hydrogen site location: inferred from neighbouring sites
*R*[*F*^2^ > 2σ(*F*^2^)] = 0.065	H-atom parameters constrained
*wR*(*F*^2^) = 0.148	*w* = 1/[σ^2^(*F*_o_^2^) + (0.058*P*)^2^ + 5.3646*P*] where *P* = (*F*_o_^2^ + 2*F*_c_^2^)/3
*S* = 1.15	(Δ/σ)_max_ < 0.001
2343 reflections	Δρ_max_ = 0.39 e Å^−^^3^
174 parameters	Δρ_min_ = −0.42 e Å^−^^3^
0 restraints	

### Special details

**Table:**

Geometry. All esds (except the esd in the dihedral angle between two l.s. planes) are estimated using the full covariance matrix. The cell esds are taken into account individually in the estimation of esds in distances, angles and torsion angles; correlations between esds in cell parameters are only used when they are defined by crystal symmetry. An approximate (isotropic) treatment of cell esds is used for estimating esds involving l.s. planes.

### Fractional atomic coordinates and isotropic or equivalent isotropic displacement parameters (Å^2^)

**Table:**

	*x*	*y*	*z*	*U*_iso_*/*U*_eq_	Occ. (<1)
Ni1	0.500000	0.20046 (3)	0.000000	0.0398 (3)	
O1	0.3442 (2)	0.16556 (13)	−0.0328 (3)	0.0482 (7)	
O2	0.5165 (3)	0.12656 (14)	0.1494 (4)	0.0516 (8)	
N1	0.3458 (3)	0.32448 (17)	−0.3947 (4)	0.0485 (9)	
N2	0.4156 (3)	0.26772 (16)	−0.1800 (4)	0.0443 (8)	
C1	0.3348 (4)	0.4311 (2)	−0.5188 (5)	0.0451 (10)	
C2	0.4275 (5)	0.4655 (3)	−0.4023 (7)	0.107 (3)	
H2	0.492752	0.442846	−0.320417	0.129*	
C3	0.2413 (4)	0.4653 (2)	−0.6326 (6)	0.0684 (15)	
H3	0.175274	0.442626	−0.712964	0.082*	
C4	0.3337 (5)	0.3562 (2)	−0.5266 (5)	0.0570 (12)	
H4A	0.261252	0.341958	−0.623726	0.068*	
H4B	0.396716	0.341706	−0.528253	0.068*	
C5	0.4370 (4)	0.28896 (19)	−0.2772 (5)	0.0435 (10)	
C6	0.3077 (4)	0.2912 (2)	−0.2387 (6)	0.0532 (12)	
H6	0.270297	0.284149	−0.193745	0.064*	
C7	0.2632 (4)	0.3262 (2)	−0.3714 (6)	0.0593 (13)	
H7	0.191169	0.347080	−0.433802	0.071*	
C8	0.5450 (4)	0.2746 (3)	−0.2605 (6)	0.0625 (13)	
H8A	0.540177	0.295302	−0.344794	0.094*	
H8B	0.611035	0.291763	−0.162159	0.094*	
H8C	0.553297	0.227662	−0.263841	0.094*	
C9	0.3562 (3)	0.06300 (18)	0.0957 (4)	0.0350 (9)	
C10	0.2234 (3)	0.06224 (19)	−0.0132 (5)	0.0437 (10)	
H10A	0.194962	0.063122	−0.121488	0.052*	
H10B	0.194416	0.101283	0.005507	0.052*	
C11	0.4003 (4)	0.06176 (19)	0.2666 (5)	0.0449 (10)	
H11A	0.374414	0.101241	0.288638	0.054*	
H11B	0.484678	0.061300	0.336412	0.054*	
C12	0.4004 (5)	0.000000	0.0664 (6)	0.0355 (12)	
H12A	0.484704	0.000000	0.135236	0.043*	
H12B	0.374053	0.000001	−0.041030	0.043*	
C13	0.1788 (5)	0.000000	0.0159 (7)	0.0460 (15)	
H13	0.093556	−0.000001	−0.054721	0.055*	
C14	0.2219 (6)	0.000000	0.1860 (8)	0.0551 (17)	
H14A	0.193036	0.038915	0.205261	0.066*	0.5
H14B	0.193036	−0.038915	0.205261	0.066*	0.5
C15	0.3539 (6)	0.000000	0.2952 (7)	0.0476 (15)	
H15	0.381400	0.000000	0.404170	0.057*	
C16	0.4075 (4)	0.12241 (18)	0.0692 (5)	0.0409 (9)	

### Atomic displacement parameters (Å^2^)

**Table:**

	*U*^11^	*U*^22^	*U*^33^	*U*^12^	*U*^13^	*U*^23^
Ni1	0.0486 (5)	0.0269 (4)	0.0458 (5)	0.000	0.0297 (4)	0.000
O1	0.0517 (17)	0.0344 (15)	0.0601 (19)	0.0081 (13)	0.0348 (16)	0.0121 (14)
O2	0.0451 (18)	0.0369 (16)	0.0623 (19)	−0.0034 (13)	0.0273 (15)	0.0097 (14)
N1	0.067 (2)	0.0355 (19)	0.044 (2)	0.0131 (17)	0.035 (2)	0.0066 (16)
N2	0.056 (2)	0.0316 (18)	0.052 (2)	0.0027 (16)	0.0361 (19)	0.0022 (16)
C1	0.057 (3)	0.037 (2)	0.037 (2)	0.0043 (19)	0.026 (2)	0.0010 (18)
C2	0.075 (4)	0.056 (3)	0.075 (4)	0.015 (3)	−0.014 (3)	0.006 (3)
C3	0.051 (3)	0.039 (2)	0.063 (3)	−0.004 (2)	0.008 (2)	−0.004 (2)
C4	0.089 (4)	0.039 (2)	0.044 (3)	0.008 (2)	0.040 (3)	0.003 (2)
C5	0.059 (3)	0.030 (2)	0.048 (2)	0.0016 (19)	0.036 (2)	0.0002 (18)
C6	0.064 (3)	0.045 (3)	0.067 (3)	0.013 (2)	0.049 (3)	0.008 (2)
C7	0.062 (3)	0.050 (3)	0.069 (3)	0.020 (2)	0.041 (3)	0.011 (2)
C8	0.066 (3)	0.061 (3)	0.073 (3)	0.010 (2)	0.049 (3)	0.018 (3)
C9	0.040 (2)	0.0280 (19)	0.038 (2)	0.0037 (16)	0.0242 (18)	0.0045 (16)
C10	0.044 (2)	0.035 (2)	0.047 (2)	0.0057 (18)	0.026 (2)	0.0032 (18)
C11	0.057 (3)	0.034 (2)	0.046 (2)	0.0007 (19)	0.033 (2)	−0.0039 (18)
C12	0.043 (3)	0.027 (3)	0.037 (3)	0.000	0.025 (3)	0.000
C13	0.032 (3)	0.046 (3)	0.056 (4)	0.000	0.024 (3)	0.000
C14	0.073 (5)	0.044 (3)	0.078 (5)	0.000	0.061 (4)	0.000
C15	0.063 (4)	0.048 (3)	0.040 (3)	0.000	0.036 (3)	0.000
C16	0.050 (3)	0.028 (2)	0.050 (2)	0.0044 (18)	0.033 (2)	0.0029 (19)

### Geometric parameters (Å, º)

**Table:**

Ni1—O1	2.179 (3)	C6—C7	1.352 (6)
Ni1—O1^i^	2.179 (3)	C7—H7	0.9300
Ni1—O2^i^	2.096 (3)	C8—H8A	0.9600
Ni1—O2	2.096 (3)	C8—H8B	0.9600
Ni1—N2^i^	2.050 (3)	C8—H8C	0.9600
Ni1—N2	2.050 (3)	C9—C10	1.528 (5)
O1—C16	1.257 (5)	C9—C11	1.534 (5)
O2—C16	1.260 (5)	C9—C12	1.540 (5)
N1—C4	1.465 (5)	C9—C16	1.526 (5)
N1—C5	1.351 (5)	C10—H10A	0.9700
N1—C7	1.364 (6)	C10—H10B	0.9700
N2—C5	1.328 (5)	C10—C13	1.530 (5)
N2—C6	1.367 (5)	C11—H11A	0.9700
C1—C2	1.345 (7)	C11—H11B	0.9700
C1—C3	1.339 (6)	C11—C15	1.535 (5)
C1—C4	1.513 (6)	C12—H12A	0.9700
C2—C2^ii^	1.395 (11)	C12—H12B	0.9700
C2—H2	0.9300	C13—H13	0.9800
C3—C3^ii^	1.403 (9)	C13—C14	1.529 (9)
C3—H3	0.9300	C14—H14A	0.9700
C4—H4A	0.9700	C14—H14B	0.9700
C4—H4B	0.9700	C14—C15	1.518 (9)
C5—C8	1.491 (6)	C15—H15	0.9800
C6—H6	0.9300		
			
O1—Ni1—O1^i^	142.26 (15)	H8B—C8—H8C	109.5
O2—Ni1—O1	61.20 (11)	C10—C9—C11	109.2 (3)
O2—Ni1—O1^i^	91.22 (12)	C10—C9—C12	108.8 (3)
O2^i^—Ni1—O1	91.22 (12)	C11—C9—C12	108.0 (3)
O2^i^—Ni1—O1^i^	61.20 (11)	C16—C9—C10	113.3 (3)
O2—Ni1—O2^i^	89.19 (18)	C16—C9—C11	109.9 (3)
N2—Ni1—O1^i^	109.38 (12)	C16—C9—C12	107.6 (3)
N2—Ni1—O1	95.55 (12)	C9—C10—H10A	109.6
N2^i^—Ni1—O1	109.38 (12)	C9—C10—H10B	109.6
N2^i^—Ni1—O1^i^	95.55 (12)	C9—C10—C13	110.1 (4)
N2—Ni1—O2^i^	91.42 (13)	H10A—C10—H10B	108.1
N2—Ni1—O2	156.75 (13)	C13—C10—H10A	109.6
N2^i^—Ni1—O2^i^	156.75 (13)	C13—C10—H10B	109.6
N2^i^—Ni1—O2	91.42 (13)	C9—C11—H11A	109.7
N2—Ni1—N2^i^	97.01 (19)	C9—C11—H11B	109.7
C5—N1—C4	127.6 (4)	C9—C11—C15	109.8 (4)
C5—N1—C7	108.1 (4)	H11A—C11—H11B	108.2
C7—N1—C4	124.3 (4)	C15—C11—H11A	109.7
C5—N2—Ni1	131.7 (3)	C15—C11—H11B	109.7
C5—N2—C6	105.9 (4)	C9—C12—C9^iii^	111.4 (4)
C6—N2—Ni1	121.6 (3)	C9^iii^—C12—H12A	109.3
C2—C1—C4	122.6 (4)	C9—C12—H12A	109.3
C3—C1—C2	117.8 (4)	C9^iii^—C12—H12B	109.3
C3—C1—C4	119.6 (4)	C9—C12—H12B	109.3
C1—C2—C2^ii^	121.1 (3)	H12A—C12—H12B	108.0
C1—C2—H2	119.4	C10—C13—C10^iii^	110.5 (5)
C2^ii^—C2—H2	119.4	C10—C13—H13	109.4
C1—C3—C3^ii^	121.1 (3)	C10^iii^—C13—H13	109.4
C1—C3—H3	119.5	C14—C13—C10	109.1 (3)
C3^ii^—C3—H3	119.5	C14—C13—C10^iii^	109.1 (3)
N1—C4—C1	113.1 (4)	C14—C13—H13	109.4
N1—C4—H4A	109.0	C13—C14—H14A	109.8
N1—C4—H4B	109.0	C13—C14—H14B	109.8
C1—C4—H4A	109.0	H14A—C14—H14B	108.3
C1—C4—H4B	109.0	C15—C14—C13	109.3 (5)
H4A—C4—H4B	107.8	C15—C14—H14A	109.8
N1—C5—C8	125.0 (4)	C15—C14—H14B	109.8
N2—C5—N1	110.1 (4)	C11—C15—C11^iii^	108.7 (5)
N2—C5—C8	124.9 (4)	C11^iii^—C15—H15	109.2
N2—C6—H6	124.9	C11—C15—H15	109.2
C7—C6—N2	110.2 (4)	C14—C15—C11	110.2 (3)
C7—C6—H6	124.9	C14—C15—C11^iii^	110.2 (3)
N1—C7—H7	127.1	C14—C15—H15	109.2
C6—C7—N1	105.7 (4)	O1—C16—Ni1	62.3 (2)
C6—C7—H7	127.1	O1—C16—O2	119.8 (4)
C5—C8—H8A	109.5	O1—C16—C9	121.8 (4)
C5—C8—H8B	109.5	O2—C16—Ni1	58.5 (2)
C5—C8—H8C	109.5	O2—C16—C9	118.4 (3)
H8A—C8—H8B	109.5	C9—C16—Ni1	167.7 (3)
H8A—C8—H8C	109.5		
			
Ni1—O1—C16—O2	11.1 (4)	C9—C10—C13—C14	60.6 (5)
Ni1—O1—C16—C9	−166.6 (3)	C9—C11—C15—C11^iii^	61.8 (6)
Ni1—O2—C16—O1	−11.6 (4)	C9—C11—C15—C14	−59.1 (5)
Ni1—O2—C16—C9	166.2 (3)	C10—C9—C11—C15	58.2 (4)
Ni1—N2—C5—N1	168.9 (3)	C10—C9—C12—C9^iii^	−58.9 (5)
Ni1—N2—C5—C8	−10.1 (6)	C10—C9—C16—Ni1	−105.9 (14)
Ni1—N2—C6—C7	−170.2 (3)	C10—C9—C16—O1	0.1 (5)
N2—C6—C7—N1	−0.3 (5)	C10—C9—C16—O2	−177.6 (4)
C2—C1—C3—C3^ii^	1.7 (7)	C10—C13—C14—C15	−60.4 (3)
C2—C1—C4—N1	63.2 (7)	C10^iii^—C13—C14—C15	60.4 (3)
C3—C1—C2—C2^ii^	−1.8 (7)	C11—C9—C10—C13	−59.4 (4)
C3—C1—C4—N1	−117.9 (5)	C11—C9—C12—C9^iii^	59.6 (5)
C4—N1—C5—N2	−179.6 (4)	C11—C9—C16—Ni1	131.7 (13)
C4—N1—C5—C8	−0.6 (7)	C11—C9—C16—O1	−122.4 (4)
C4—N1—C7—C6	179.8 (4)	C11—C9—C16—O2	59.9 (5)
C4—C1—C2—C2^ii^	177.1 (3)	C12—C9—C10—C13	58.2 (5)
C4—C1—C3—C3^ii^	−177.2 (3)	C12—C9—C11—C15	−60.0 (5)
C5—N1—C4—C1	−111.5 (5)	C12—C9—C16—Ni1	14.4 (15)
C5—N1—C7—C6	0.0 (5)	C12—C9—C16—O1	120.3 (4)
C5—N2—C6—C7	0.4 (5)	C12—C9—C16—O2	−57.4 (5)
C6—N2—C5—N1	−0.4 (5)	C13—C14—C15—C11	60.0 (3)
C6—N2—C5—C8	−179.4 (4)	C13—C14—C15—C11^iii^	−60.0 (3)
C7—N1—C4—C1	68.7 (6)	C16—C9—C10—C13	177.8 (4)
C7—N1—C5—N2	0.2 (5)	C16—C9—C11—C15	−177.0 (4)
C7—N1—C5—C8	179.2 (4)	C16—C9—C12—C9^iii^	178.1 (3)
C9—C10—C13—C10^iii^	−59.4 (6)		

Symmetry codes: (i) −*x*+1, *y*, −*z*; (ii) *x*, −*y*+1, *z*; (iii) *x*, −*y*, *z*.
